# Antifungal Activity of Hop Leaf Extracts and Xanthohumol on Two Strains of *Venturia inaequalis* with Different Sensitivities to Triazoles

**DOI:** 10.3390/microorganisms11061605

**Published:** 2023-06-17

**Authors:** Sophie Moureu, Justine Jacquin, Jennifer Samaillie, Caroline Deweer, Céline Rivière, Jérôme Muchembled

**Affiliations:** Joint Research Unit 1158 BioEcoAgro, INRAE, University of Lille, JUNIA, University of Liège, UPJV, University of Artois, ULCO, 59000 Lille, France; sophie.moureu@gmail.com (S.M.); justine.jacquin@univ-lille.fr (J.J.); jennifer.samaillie@univ-lille.fr (J.S.); caroline.deweer@junia.com (C.D.)

**Keywords:** *Venturia inaequalis*, triazole sensitivity, *Humulus lupulus* L., antifungal activity, bioguided fractionation, xanthohumol

## Abstract

Hop cones are well-known for their antimicrobial properties, attributed to their specialized metabolites. Thus, this study aimed to determine the in vitro antifungal activity of different hop parts, including by-products such as leaves and stems, and some metabolites against *Venturia inaequalis*, the causal agent of apple scab. For each plant part, two types of extracts, a crude hydro-ethanolic extract and a dichloromethane sub-extract, were tested on spore germination of two strains with different sensitivities to triazole fungicides. Both extracts of cones, leaves and stems were able to inhibit the two strains, whereas rhizomes did not show activity. The apolar sub-extract of leaves appeared as the most active modality tested with half maximal inhibitory concentrations (IC_50_) of 5 and 10.5 mg·L^−1^ on the sensitive strain and the strain with reduced sensitivity, respectively. Differences in activity level between strains were noticed for all active modalities tested. Sub-extracts of leaves were then separated into seven fractions by preparative HPLC and tested on *V. inaequalis*. One fraction, containing xanthohumol, was especially active on both strains. This prenylated chalcone was then purified by preparative HPLC and showed significant activity against both strains, with IC_50_ of 1.6 and 5.1 mg·L^−1^. Therefore, xanthohumol seems to be a promising compound to control *V. inaequalis*.

## 1. Introduction

*Venturia inaequalis* (Cooke) G.Winter (sexual phase), also named *Fusicladium pomi* (Fr.) Lind (asexual phase), is a hemibiotrophic ascomycete fungus responsible for apple scab [1]. Apple scab is a widespread disease found in all apple-growing regions [2]. Also named black spot, it is the most economically important disease in apple orchards, causing huge crop losses, with up to 70% reduction in apple production [3,4]. Scab infection causes visible lesions on leaves and fruits, leading to repeated defoliation and unmarketable fruits. This disease is very problematic from an economic and environmental point of view due to the use of phytosanitary products, since it requires up to 25 treatments per year, depending on the weather [5,6].

Even though integrated practices, such as prophylactic methods and genetics, are used to manage *V. inaequalis*, fungicide treatments remain the main practice used. This intensive use of single-site chemical fungicides leads to the appearance of a resistance phenomenon towards several families. *V. inaequalis* is considered as a high-risk pathogen for developing resistance to fungicides [7]. The first cases of resistance were observed in the 1960s and 1970s, with both dodine and the MBC (Methyl Benzimidazole Carbamate) class [8]. Since the 1980s, two other classes of fungicides have been used, demethylation inhibitors (DMIs) and quinone-outside inhibitors (QoI). Resistance to the DMI class is well-known worldwide, with the first event in commercial conditions reported in 1995 [9]. Copper- and sulphur-based products are used in organic farming but also lead to phytotoxic and environmental problems [10]. Thus, the interest in biopesticides, to some extent botanical pesticides, is growing [11]. Despite the emphasis placed on the search for new alternatives for crop protection, including the potential of plant extracts or molecules derived from these extracts, only a few products have been approved.

Hop (*Humulus lupulus* L., Cannabaceae) could represent a plant with a high antifungal potential thanks to its specialized metabolites synthesized by lupulin glands, found in female inflorescences (hop cones) and to a lesser extent in leaves. Hop cones are indeed composed of prenylated phenolic compounds and terpenoids, already recognized as active metabolites in many fields. Due to the aromatic, bittering and preserving properties of cones, hop is mostly cultivated for the brewing industries. However, it is also a well-known medicinal plant, as many biological properties have been attributed to it. For instance, studies have shown that *H. lupulus* may have an inhibitory effect on certain cancers, could be an alternative to treat menopause symptoms or can exhibit direct antioxidant activities [12,13,14]. Hop is also well known for its antimicrobial activities against human pathogens, including antibacterial properties against numerous bacteria [15,16,17], as well as antifungal activities against dermatophytic fungi such as *Trichophyton* spp. [18].

In recent years, hop has attracted great interest for its potential application in food and crop protection, as a food preservative or against pathogens and insect pests. Toxic or repellent activities have been attributed to hop metabolites or essential oil against various pests such as *Drosophila suzukii* [19], *Sitophilus granarius* [20] and *Varroa destructor* [21]. More and more studies have focused on the antifungal or anti-oomycete activities of hop. Cone essential oil was found to be active against the wheat pathogen *Zymoseptoria tritici* [22]. Moreover, hop extracts have also successfully inhibited mycelial growth or spore germination of numerous pathogens including *Fusarium* spp., *Aspergillus* spp., *Penicillium* spp., *Botrytis cinerea* and *Phytophthora infestans* [23,24,25,26,27,28].

These biological properties are usually attributed to hop cones and their metabolites, which are already widely used for beer production. However, other parts of the crop, such as hop leaves and stems, remain very little exploited [29,30]. Thus, there is an interest in researching the potential use of these waste materials to add value to the crop.

**Figure 1:**
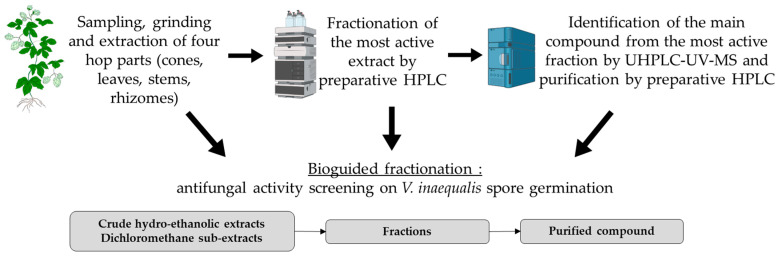
Schematic research strategy.

The aim of the present study was to evaluate the in vitro antifungal properties of hop against the phytopathogen *Venturia inaequalis*. Thus, two extractions were carried out on four hop parts (cones, leaves, stems and rhizomes) which made it possible to obtain a crude hydro-ethanolic extract and a dichloromethane sub-extract for each part. These extracts were screened and compared on spore germination of two strains of *V. inaequalis* having different sensitivities to tebuconazole, a fungicidal agent from the triazole family. The extract which proved to be the most active on the two strains was fractioned into seven fractions by preparative HPLC. The fractions were then screened for their antifungal activity, and the chalcone purified from the most active fraction, xanthohumol, was tested (Figure 1).

## 2. Materials and Methods

### 2.1. Hop Phytochemistry

#### 2.1.1. Preparation of Extracts from 4 Hop Parts

Female hop plants (Nugget cultivar) were collected at the maturity stage in French Flanders (Beck farm, Bailleul, France). A voucher specimen was kept at the Faculty of pharmacy in Lille (laboratory of pharmacognosy) under reference NugBeck2019. Each part of the plant (cones, leaves, stems and rhizomes) was separated and dried for ten days at room temperature. Then, crude hydro-ethanolic extracts (CHE) and dichloromethane sub-extracts (DSE) of each part of the plant were obtained as previously described in Jacquin et al. [28]. Ultra-pure water was obtained using a Millipore Integral 5 (Merck™, Trosly-Breuil, France) water purification system with a resistivity of not less than 18 MΩ·cm^−1^, whereas ethanol and dichloromethane were purchased from VWR Prolabo^®^ (Fontenay-sous-Bois, France). Briefly, crude hydro-ethanolic extracts (CHE) were obtained by four successive macerations stirred in the dark with ethanol/water (9:1, *v*/*v*). Ethanol was evaporated with a rotary evaporator (HeidolphTM, Schwabach, Germany), and the CHE were then freeze-dried (Telstar CryodosTM, Barcelona, Spain). CHE were then subjected to a liquid/liquid extraction with water/dichloromethane (DCM) (5:5, *v*/*v*). After addition of anhydrous sodium sulfate (Na_2_SO_4_) and filtration, DCM (VWR Prolabo^®^) was evaporated to obtain a DCM sub-extract (DSE) for each part. The percentage yields for each crude extract and for each DCM sub-extract (cones, leaves, stems and rhizomes) were, respectively, 25.6%, 8.8%, 8.4% and 16.8% and 39%, 31%, 17.6%, 7.7% on a dry weight basis.

#### 2.1.2. Fractionation of Hop Leaf DSE by Preparative HPLC

Hop leaf DSE was fractionated by preparative HPLC using a Shimadzu^®^ HPLC system equipped with a LC-20AP binary high-pressure pump, a CBM-20A controller and an SPD-M20A diode array detector. The software used was LabSolution version 5.3. Five hundred µL of a filtered solution of leaf DSE at 100 mg·mL^−1^, solubilized in methanol, were injected into an Uptisphere Strategy C18-HQ (250 × 21.2 mm, 5 μm,) prep-LC column (Interchim, France). The mobile phases consisted of two solvents with the addition of 0.1% (*v*/*v*) formic acid: ultra-pure water (A) and acetonitrile (B). Acetonitrile (LC-MS grade) was purchased from Carlo Erba Reagents^®^ (Val-de-Reuil, France) and formic acid from Merck™ (Darmstadt, Germany). The following gradient was applied: 10–100% B (0–45 min), then 100% B (45–65 min). The flow was maintained at 16 mL·min^−1^. Seven fractions, obtained from seventeen successive injections, were pooled according to their chromatographic profiles: F1 (0–10 min), F2 (10–20 min), F3 (20–30 min), F4 (30–40 min), F5 (40–46.5 min), F6 (46.5–52.5 min) and F7 (52.5–65 min). Each fraction was analysed using UHPLC-UV-MS. The overall purification yield was approximately 37.4% with F1 6.1%, F2 3.3%, F3 2.8%, F4 3.6%, F5 4.1%, F6 5.6% and F7 12.0%, respectively.

#### 2.1.3. Purification of Xanthohumol from Hop Leaf DSE

Xanthohumol detected in fraction F4 of hop leaf DSE was partially purified by preparative HPLC using the equipment, the column and the software described in Section 2.1.2. Five hundred µL of a filtered solution of leaf DSE at 100 mg·mL^−1^ in methanol were injected 13 times (total mass injected = 635 mg). The mobile phases consisted of water (A) and acetonitrile (B), with 0.1% (*v*/*v*) formic acid. Elution was performed as follow: 30% B (0–2.5 min), 30–55% B (2.5–10 min), 55% B (10–35 min), 55–100% B (35–40 min), 100% B (40–55 min) with a flow rate of 15 mL·min^−1^. Xanthohumol was obtained and its purity was analysed by UHPLC-UV-MS. Its yield was approximately 0.7% (4.49 mg).

#### 2.1.4. Purification of Xanthohumol from Hop Cone DSE

Xanthohumol was purified from hop cones as previously described in Bocquet et al. [31]. Briefly, this chalcone was purified from the DCM sub-extract of cones by CPC using the Arizona solvent system P: n-heptane/EtOAc/MeOH/water (6:5:6:5; *v*/*v*) in the ascending mode. After 60 min in ascending mode, the CPC then switched to extrusion mode for 10 additional minutes. The fractionation was monitored by online UV absorbance measurements at 254 nm and 370 nm. Based on TLC developed with toluene/ethyl acetate/formic acid (73:18:9; *v*/*v*), fractions were pooled into 5 sub-fractions (MC1 to MC5) from ascendant mode and 3 sub-fractions (MC6 to MC8) from extrusion mode. This CPC method allowed us to purify, in one step, xanthohumol with 98% purity from MC4.

#### 2.1.5. Structural Identification of Xanthohumol from Hop Cones by NMR

Nuclear Magnetic Resonance (NMR) spectra were recorded on a Bruker^®^ DPX-500 spectrometer.

1H-NMR spectrum (500 MHz, MeOD): δ (ppm) 7.81 (CH, d, J = 15.4 Hz, H-α), 7.69 (CH, d, J = 15.4 Hz, H-β), 7.52 (CH, d, J = 8.6 Hz, H-2 and H-6), 6.84 (CH, d, J = 8.6 Hz, H-3 and H-5), 6.03 (CH, s, H-5′), 5.22 (CH, t, J = 7.3 Hz, H-2″), 3.91 (OCH3, s), 3.25 (CH2, d, J = 7.3 Hz, H-1″), 1.78 (CH3, s, H-5″), 1.67 (CH3, s, H-4″) and 13C-NMR spectrum (500 MHz, MeOD): 192.5 (C=O), 162.2 (C-4′), 161.3 (C-2′), 160.3 (C-6′), 159.5 (C-4), 142 (C-β), 129.9 (C-3″), 129.9 (C-2), 129.9 (C-6), 127 (C-1), 124 (C-α), 122.8 (C-2″), 114.8 (C-3), 114.8 (C-5), 107.9 (C-3′), 104.2 (C-1′), 89.3 (C-5′), 54.4 (OCH3), 24.3 (C-4″), 20.5 (C-1″), 16.1 (C-5″).

#### 2.1.6. UHPLC-UV-MS Analysis

Ultra-High Performance Liquid Chromatography (UHPLC) analyses were carried out using an Acquity UPLC^®^ H-Class Waters^®^ system (Waters, Guyancourt, France) equipped with two independent pumps, a diode array detector (DAD) and an Acquity QDa ESI-Quadrupole Mass Spectrometer. The software used was Empower 3. The stationary phase was a Waters^®^ Acquity BEH C18 column (2.1 × 50 mm, 1.7 μm) connected to a 0.2 µm in-line filter. The mobile phase was composed of (A) ultrapure water + 0.1% formic acid and (B) acetonitrile + 0.1% formic acid. Three methods were used for analysis: (1) Flow rate and column temperature were set at 0.45 mL·min^−1^ and 40 °C ± 5 °C, respectively. Fractions and compounds were eluted using the following gradient: 10% B (0–1 min), 10–75% B (1–3 min), 75% B (3–5 min), 75–100% B (5–7 min) and 100% B (7–9 min) before column re-equilibration for 2 min; (2) Flow rate and column temperature were set at 0.3 mL·min^−1^ and 30 °C ± 5 °C, respectively. Fractions and compounds were eluted using the following gradient: 30% B (0–0.5 min), 30–48% B (0.5–2 min), 48% B (2–6 min), 48–100% B (6–7.5 min) and 100% B (7.5–8 min) before column re-equilibration for 2 min; (3) Flow rate and column temperature were set at 0.3 mL·min^−1^ and 30 °C ± 5 °C, respectively. Fractions and compounds were eluted using the following gradient: 50% B (0–1 min), 50–75% B (1–3 min), 75% B (3–5 min), 75–100% B (5–7 min) and 100% B (7–9 min) before column re-equilibration for 3 min. For the three methods, the other parameters were similar. The wavelength range was fixed at (200–790) nm with a resolution of 1.2 nm. Ionization was carried out in both negative and positive mode with a mass range of 100 to 1000 Da. Cone voltage value was 15 V in positive mode and 30 V in negative mode, capillary voltage value was 0.8 kV and probe temperature was 600 °C. The injection volume was set at 2 µL. All samples were prepared at 0.1 mg·mL^−1^ or 1 mg·mL^−1^ in analytical grade MeOH. 

### 2.2. Antifungal Activity against Two Strains of Venturia inaequalis

#### 2.2.1. Culture Conditions and Inoculum Preparation

Two strains with different sensitivities to tebuconazole, an active substance from the triazole family, were used for these experiments. The strains were previously characterized as sensitive (S755) and with reduced sensitivity (rs552) to tebuconazole [32]. They were both provided by the IRHS ECOFUN team from INRAE Angers-Nantes centre (France). Spore suspensions were obtained from a 20-day-old culture maintained on malt agar medium at 20 °C in the dark. Spores were collected in glucose–peptone medium (14.3 g of glucose and 7.1 g of bactopeptone per litre) and suspensions were calibrated at 5 × 10^4^ spores·mL^−1^.

#### 2.2.2. In Vitro Assays

The activity of hop extracts, fractions from leaf DSE, purified xanthohumol and fungicide-active substances (triazoles and copper sulphate) were evaluated on spore germination on liquid medium, using 96-well plates (Corning^®^ 3595, Corning Incorporated, Somerville, MA, USA), with a protocol adapted from Muchembled et al. [32]. Each product was dissolved in dimethyl sulfoxide (DMSO) (Sigma Aldrich, Saint-Quentin-Fallavier, France) and then added to the glucose–peptone medium. Briefly, the protocol consisted of filling 112 µL per well, one concentration per line of six wells corresponding to half-microplates. Thereafter, 38 µL of glucose–peptone were added to the first two wells of each line, used as a control for net optical density (OD). The same volume of calibrated spore suspension was used to fill the four other wells of the line, corresponding to four replicates per concentration. Plates were sealed and left for six days at 20 °C in the dark with shaking at 140 rpm. First, the sensitivity of the two strains to tebuconazole and dife-noconazole (Sigma Aldrich, Saint-Quentin-Fallavier, France) was evaluated. Concerning hop extracts, eight concentrations between 2 and 1000 mg·L^−1^ were tested for CHE and DSE, chosen according to the modality. Copper sulphate (CuSO4, Merck^®^, Darmstadt, Germany) was used as the reference mineral fungicidal active substance and tested at a range of concentrations between 8 and 500 mg·L^−1^. According to the results of extracts and sub-extracts, fractions from leaf DSE were screened at four concentrations between 0.125 and 125 mg·L^−1^. The active fractions were then tested at concentrations from 0.06 to 37 mg·L^−1^ for F4 and from 5 to 280 mg·L^−1^ for F5. Finally, the activity of xanthohumol, a purified chalcone from the most active fraction, was determined, with concentrations tested from 0.06 to 125 mg·L^−1^.

#### 2.2.3. Data Analyses

Optical density (OD) was read at 630 nm after 6 days of incubation with a spectrophotometer (Biotek EL 808, BioTek Instruments, Santa Clara, CA, USA). Once the net optical density was calculated, half inhibitory concentration (IC_50_), the concentration that inhibits the spore germination by 50%, was determined using a non-linear regression with four parameters (dose-response curve). Each experiment was repeated at least twice to obtain one IC_50_ per modality taking into account inter-and intra-experiments variability. A Fisher test with a *p*-value (α = 5%) was first performed to compare all the modalities. Afterwards, a pairwise comparison of IC_50_, based on 95% confidence intervals with Bonferroni adjustments, was used for each strain separately, and then for each modality, comparing the two strains. Statistical analyses were performed using R-software (rCore Team, 2019) and the nlstools package.

## 3. Results

### 3.1. Characterization of Strain Sensitivity to Triazoles and Copper Sulphate

Fungicide active substances from the triazole family were tested on the two strains: tebuconazole and difenoconazole. The results obtained showed that the S755 strain was more sensitive than rs552 to the two triazoles tested (Table 1). Interestingly, there was also a difference between the two strains with copper sulphate but at the opposite of the triazole sensitivity. Rs552 appeared to be more sensitive to copper sulphate than S755.

### 3.2. Screening of Hop Crude Extracts and Apolar Sub-Extracts

**Figure 2:**
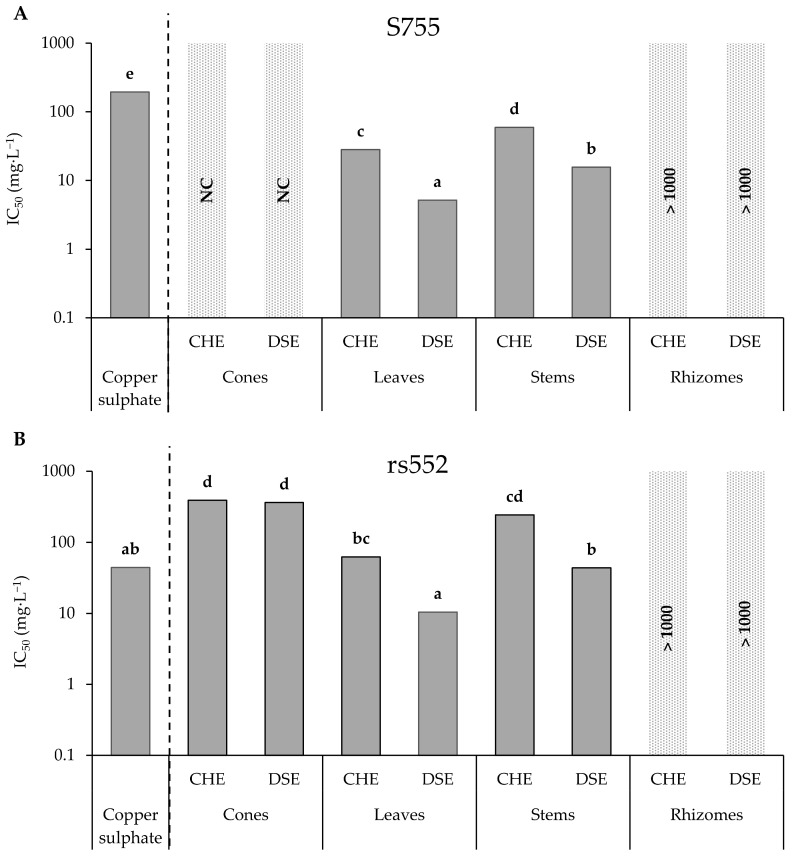
IC_50_ values of extracts from four hop parts on *V. inaequalis* spore germination. Different letters correspond to significant differences. NC = Not Calculable IC_50_; IC_50_ > 1000 mg·L^−1^ = Not active.

For the strain S755, at least two parts of the hop plant showed strong antifungal activity with calculable IC_50_ for leaves and stems (Figure 2A). Leaf and stem DSE, with IC_50_ of 5.2 and 15.6 mg·L^−1^, respectively, were more active than their respective CHE (IC_50_ of 28.2 and 59.3 mg·L^−1^). The leaf DSE was statistically the most active sub-extract tested. Regarding CHE and DSE of cones, IC_50_ were not calculable, because both extracts only slightly inhibited spore germination at the highest concentration tested. Finally, no activity was recorded for the CHE and the DSE of rhizomes. For copper sulphate, an IC_50_ of 194.6 mg·L^−1^ was determined. Thus, leaf and stem extracts were more active than copper sulphate for this strain.

For the strain rs552, antifungal activity was recorded for three of the four hop parts (Figure 2B). The leaf DSE was also considered as the most active sub-extract tested on this strain, with an IC_50_ of 10.5 mg·L^−1^. Again, leaf and stem DSE (43.7 mg·L^−1^) were more active than their respective CHE with IC_50_ of 62.5 and 242.1 mg·L^−1^, respectively. It should be noted that IC_50_ were calculable for cone extracts (389.7 mg·L^−1^ for CHE and 361.6 mg·L^−1^ for DSE) on this strain but showed lower antifungal activity than leaves and stems. No activity was recorded for the CHE and the DSE of rhizomes. Copper sulphate, on this strain, showed an IC_50_ of 44.4 mg·L^−1^ and no statistical difference with leaf and stem DSE was noticed.

### 3.3. Bioguided Fractionation of Leaf DSE

**Figure 3:**
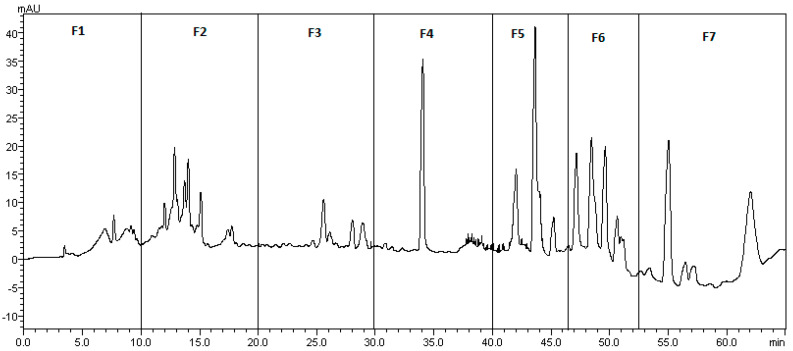
Fractionation of hop leaf DSE by preparative HPLC. Chromatogram obtained at 330 nm. F1: 0–10 min; F2: 10–20 min; F3: 20–30 min; F4: 30–40 min; F5: 40–46.5 min; F6: 46.5–52.5 min and F7: 52.5–65 min.

The leaf DSE, appearing as the most active sub-extract on the two strains, was fractionated by HPLC preparative into seven fractions (Figure 3).

**Figure 4:**
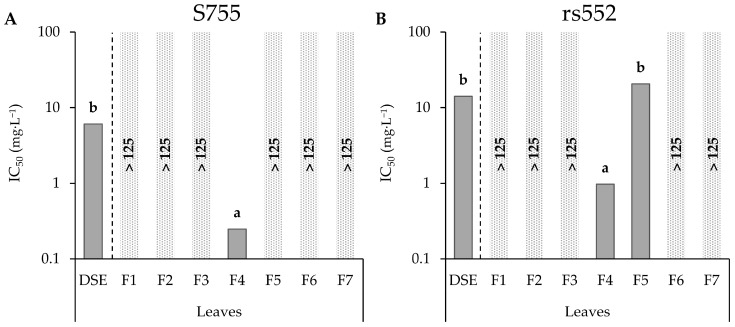
IC_50_ values of fractions from leaf DSE on the two strains of *V. inaequalis*. Different letters correspond to significant differences. IC_50_ > 125 mg·L^−1^ = Not Active.

A screening of all fractions obtained from leaf DSE (Supplementary Materials, Figure S1) at a maximum concentration of 125 mg·L^−1^ showed that five of them (F1, F2, F3, F6 and F7) did not inhibit spore germination of the two *V. inaequalis* strains (Figure 4). Regarding the S755 strain, only one fraction, F4, appeared to be highly active with an IC_50_ of 0.25 mg·L^−1^ (Figure 4A). This fraction was statistically more active than the leaf DSE. For the strain rs552, F4 was also the most active fraction with an IC_50_ of 0.97 mg·L^−1^ (Figure 4B). Unlike S755, rs552 seemed to be sensitive to F5 with calculable IC_50_ of 21 mg·L^−1^. F5 appeared to be as active as leaf DSE.

**Figure 5:**
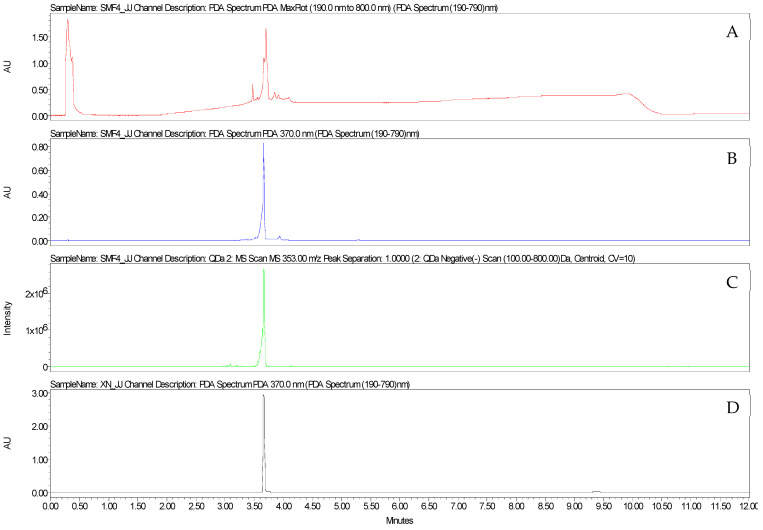
Chromatograms obtained by UHPLC-UV-MS (method 1) of F4 (**A**) MaxPlot, (**B**) 370 nm, (**C**) negative ion mode ESI-MS of the [M-H]^−^ at *m*/*z* 353) and (**D**) xanthohumol purified from hop cones (370 nm).

**Figure 6:**
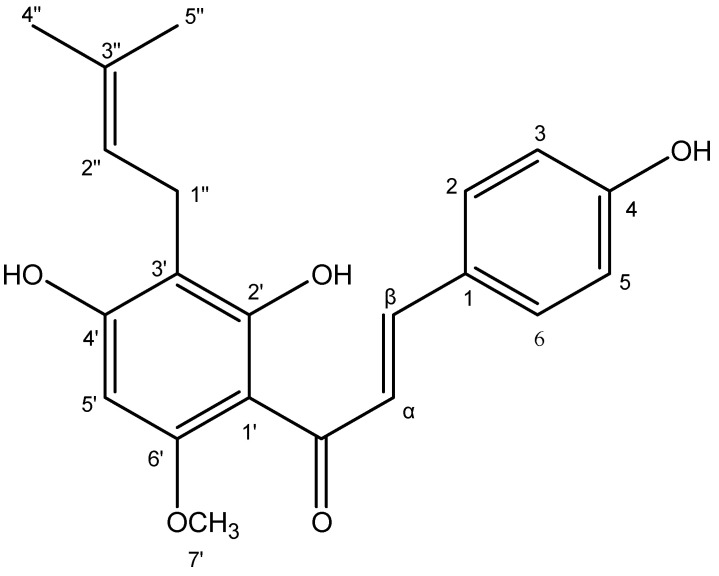
Structure of xanthohumol.

**Figure 7:**
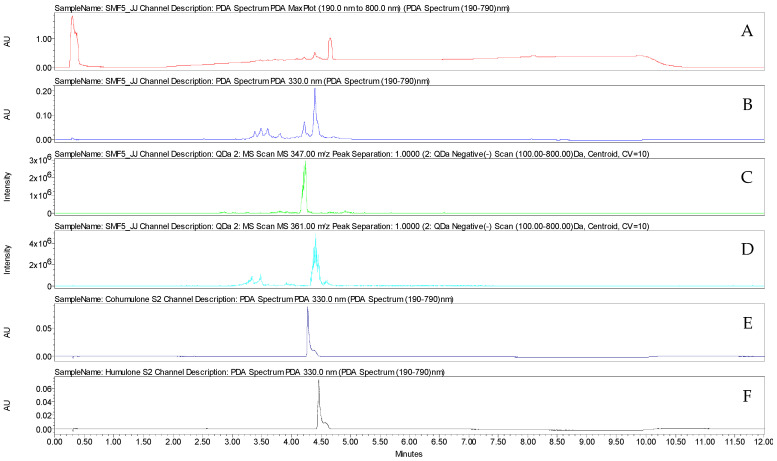
Chromatograms obtained by UHPLC-UV-MS (method 1) of F5 (**A**) MaxPlot, (**B**) 330 nm, (**C**) negative ion mode ESI-MS of the [M-H]^−^ at *m*/*z* 347 and (**D**) negative ion mode ESI-MS of the [M-H]^−^ at *m*/*z* 361) and (**E**) co-humulone and (**F**) humulone purified from hop cones (330 nm).

The fraction F4 was analysed by UHPLC-UV-MS. The major compound identified in this fraction was xanthohumol, with an estimated purity of 62.53% (Supplementary Materials, Figures S2 and S3). The identification of this prenylated chalcone was confirmed based on the comparison with the retention time and UV and mass spectra of xanthohumol purified in the laboratory from hop cones (Figure 5 and Figure 6). The F5 fraction is mainly made up of alpha acids (Figure 7).

**Figure 8:**
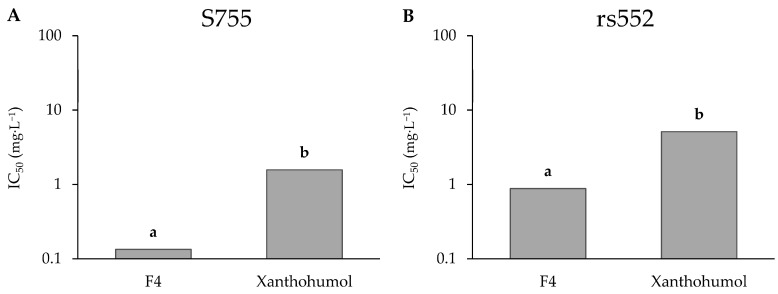
IC_50_ values of xanthohumol purified from leaves on both strains of *V. inaequalis*. Different letters correspond to significant differences.

Thus, xanthohumol was purified from leaf DSE by preparative HPLC and tested on both strains. Its purity after purification is estimated at 99% (Supplementary Materials, Figures S4 and S5). It appeared to be very active on the two strains with IC_50_ of 1.6 and 5.1 mg·L^−1^ against S755 and rs552, respectively (Figure 8). The activity of xanthohumol, purified at 98% from hop cones and identified by NMR (Supplementary Materials, Figure S6), was tested. With a similar purity, it also showed high activity with IC_50_ of 2.8 and 7.4 mg·L^−1^ against S755 and rs552, respectively (data not shown). However, even though xanthohumol was the main compound of fraction 4, it was statistically less active than this fraction.

### 3.4. Comparison of the Strain Sensitivities to Hop Extracts, Fraction and Xanthohumol

Regarding hop extracts and sub-extracts, a difference in sensitivity between the strains was recorded for cones, leaves, and stems (Table 2). Rs552 can be considered more sensitive than S755 to cone extracts because these extracts slightly inhibited S755, and IC_50_ could not be determined. For other extracts tested, IC_50_ were statistically different between the strains. Unlike cone extracts, a significant difference was noticed for leaves and stems, with S755 being more sensitive than rs552. No difference could be noticed only for rhizomes extracts, since they were not active on the two strains of *V. inaequalis*.

No difference between strains was noted with F1, F2, F3, F6 and F7, as these fractions were not active on S755 nor on rs552. However, the strains seemed to have different sensitivities to two fractions, F4 and F5 (Table 2). S755 was more sensitive to the most active fraction, F4. By contrast, F5 had an antifungal activity against rs552 and no activity against S755.

Finally, concerning xanthohumol extracted from leaves, the S755 strain was more sensitive than rs552.

## 4. Discussion

### 4.1. Different Sensitivities in V. inaequalis Strains Are Noticed with Compounds of Plant Origin

This study focused on two strains of *V. inaequalis* that were previously characterized as sensitive and with reduced sensitivity to tebuconazole [32]. Tebuconazole, a triazole fungicide, belongs to the demethylation inhibitor family (DMI) that acts on the 14α-demethylase related to the CYP51 gene [33]. Many cases of resistance to DMI fungicides appeared on *V. inaequalis* and other phytopathogens strains [34,35,36]. The difference of sensitivities between the two strains was confirmed in this study not only with tebuconazole but also with difenoconazole. Although the resistance mechanism for rs552 has not been characterized yet, we can discuss three main mechanisms identified to explain the DMI resistance. The most widely reported resistance mechanism is the presence of a point mutation on the CYP51 gene, decreasing the affinity between the target and the substance, thus leading to triazole tolerance. The CYP51 gene could also be overexpressed, increasing the amount of 14α-demethylase in the cells, leading to a reduction of DMI sensitivity. The third resistance mechanism is the overexpression of efflux pump genes as ATP binding cassette (ABC) or major facilitator superfamily (MFS) transporters, making it possible to decrease the accumulation of intracellular substance by releasing it outside the cell [33,37].

Interestingly, copper sulphate seemed to act in opposition to the triazole resistance for these strains, because rs552 was more sensitive than S755. The copper-based products are indeed multi-site contact fungicides (M01–FRAC) and have a broad-spectrum activity. There are mentions of resistance cases mainly on bacteria but also some tolerance in fungi [38]. Two types of mechanisms are involved in heavy metal detoxification, including of copper, in fungi [39]. The first potential mechanism is the secretion of metabolites, which could bind to the metals directly on the extracellular space but also on the cell wall, resulting in their inactivation. The second mechanism is metal chelation when the substance enters the cell, leading to inactivation and storage of metals away from sensitive metabolic processes. Thus, efflux pumps could also be involved in copper detoxification [40].

Differences in sensitivity between the two strains were also observed with active hop extracts and compounds. On one hand, as triazole sensitivity, S755 was more sensitive to hop leaf and stem extracts, fraction 4 of leaf DSE and xanthohumol than rs552. On the other hand, rs552 was more sensitive to hop cone extracts and fraction 5 of leaf DSE than S755. Thus, these results suggest that different strains of the same pathogen could have different responses to compounds of plant origin. This difference could be linked to genetic diversity. On these strains, rs552 already appeared to be more sensitive than S755 to several essential oils [32]. Furthermore, these differences in responses to hop essential oil have already been noticed by Jiang et al. [26] on two strains of *F. graminearum*.

As plant extracts and essential oils are complex mixtures of metabolites, it is difficult to determine which molecule may be responsible for higher activity against one strain over the other. However, in this study, S755 was determined to be more sensitive to xanthohumol, whereas rs552 seemed more sensitive to α-acids.

### 4.2. Antifungal Properties of Hop on V. inaequalis

Besides the use of hop cones by the brewing industry, hop is a source of biologically active metabolites. Among the properties attributed, hop appears to be active on fungi but its use for the control of phytopathogens remains under-studied.

Thus, the potential antifungal properties of different hop parts against *V. inaequalis*, the apple scab agent, were first investigated in this study. It revealed that hop can inhibit spore germination of *V. inaequalis*. On the eight extracts tested (CHE and DSE of four hop parts), six of them showed an antifungal activity against at least one strain. Only hop rhizomes were not active on *V. inaequalis*. Previously, cones proved to be effective on fungi thanks to metabolites synthesized by lupulin glands at the base of the bracts. Hop cone composition is much studied in the literature, as the interest in this crop has grown in the last years. Many factors can influence the concentration of metabolites in cones, such as the cultivar, growth conditions, and also the location of the culture [41]. The extracts tested in this study were previously analysed and showed that cones were mainly composed of bitter acids, α- and β-acids, and xanthohumol [28]. Cone extracts, spent hops or cone essential oil were already identified as being active against other field or storage fungi, with activity close to the one observed on *V. inaequalis.* Cone extracts were successful in inhibiting mycelial growth or spore germination of various fungi such as *Fusarium spp*, *Botrytis cinerea, Epicoccum nigrum, Alternaria alternata, Rhizoctonia solani, Sclerotinia sclerotiorum* and *Zymoseptoria tritici*, with inhibition ranging from 20% to 85% [22,27,42,43]. Cone essential oil also appeared to be active on *Z. tritici* and *F. graminearum* growth, with IC_50_ of 360 mg·L^−1^ and EC_50_ of 7 mg·g^−1^, respectively [22,26]. Furthermore, hop essential oil showed an inhibitory effect on mycotoxin production of *F. graminearum* [26]. Bocquet et al. [22] also tested extracts of different hop parts, but extracts of leaves, stems and rhizomes were only slightly active on *Z. tritici*, in comparison with cone extracts (IC_50_ = 0.73 g·L^−1^ for the hydro-ethanolic crude extract). The authors identified co-humulone and desmethylxanthohumol as active compounds against this pathogen. Interestingly, cone extracts were not the most active extracts against *V. inaequalis.* Leaf and stem extracts, more especially DSE, were more active than copper sulphate, a fungicidal active substance used in organic agriculture, or had similar activity.

### 4.3. Hop Leaves, a Promising Source of Antifungal Agents?

Currently, in hop culture, only female inflorescences are valorised for beer production, or to a lesser extent for medicinal properties. However, leaves and stems represent almost 75% of crop biomass and are poorly exploited to date [29,30,44,45]. Finding a use for these by-products, usually considered as waste, would be an added value for producers. In recent years, some studies have focused on leaf and stem composition but also on their potential use. For instance, Afonso et al. [29] underlined the nutrient richness of the leaves that can be used in composting mixtures. Natural cellulose fibres could be obtained from hop stems [45]. Moreover, antimicrobial properties were attributed to leaves. Abram et al. [15] tested the antibacterial potential of hop leaf extracts and showed that they had relatively close activities against gram positive bacteria, *Staphylococcus aureus*, and gram negative bacteria, *Escherichia coli* (0.16 mg·mL^−1^ < MIC < 0.48 mg·mL^−1^). In the present study, leaf extracts, in particular DSE, appeared as the most promising extracts to inhibit the spore germination of *V. inaequalis* with IC_50_ values of 5 and 10 mg·L^−1^ depending on the strain tested. Leaves already appeared as a promising hop part to inhibit the spore germination and the mycelial growth of one oomycete, *Phytophthora infestans* [28]. Therefore, hop leaf extracts appear to be promising extracts for controlling different plant pathogens.

Regarding leaf composition, the content of prenylated chalcones (xanthohumol and desmethylxanthohumol) and bitter acids has already been studied and depends on the cultivar [46,47,48]. Xanthohumol and bitter acids were previously quantified in leaves [28] and their presence is confirmed in this study in different fractions of DSE from Nugget cultivar leaves. Bioguided fractionation made it possible to identify the fraction containing xanthohumol as the most active one against *V. inaequalis*. The fraction containing α-acids also showed antifungal activity against one strain, compared to the fraction with β-acids which was not active on both strains. Thus, as previously reported, humulone and its derivatives appeared to be more active than lupulone and its derivatives against fungi [22,49].

### 4.4. The Case of Xanthohumol: A Metabolite with Interesting Antimicrobial Properties

Xanthohumol is mainly synthesized in hop cones, but less attention is paid to hop leaves, from which this chalcone can also be synthesized, and which represents a significant part of hop biomass. Many antimicrobial properties have been attributed to hop prenylated chalcones (xanthohumol, desmethylxanthohumol) and flavanones (isoxanthohumol). For human health, xanthohumol and desmethylxanthohumol were reported to be able to inhibit the growth of methicillin-resistant *Staphylococcus aureus* with MIC of 9.8 mg·L^−1^ and 19.5 mg·L^−1^, respectively [31]. Moreover, xanthohumol was active on *Trichophyton* spp. and slightly active on *Mucor rouxianus* whereas isoxanthohumol was only slightly active on *Trichophyton mentagrophytes* [18]. These compounds can also find a potential application for the control of plant and post-harvest fungi. For example, isoxanthohumol was particularly active on *Botrytis cinerea, Sclerotinia sclerotiorum* and *Fusarium graminearum* with IC_50_ of 4.3, 14.5 and 16.5 mg·L^−1^, respectively [27]. Moreover, xanthohumol was able to slightly inhibit mycelial growth and spore germination of *Phytophthora infestans* [28]. It also showed activity against three *Fusarium* species (*culmorum, semitectum and oxysporum*) with MIC_50_ ranging from 15 to 100 mg·L^−1^ [24]. Interestingly, xanthohumol was not active on *Z. tritici*, but its precursor, desmethylxanthohumol, was one of the most active purified compounds with an IC_50_ value of 200 mg·L^−1^ [22]. In addition to these antifungal properties already demonstrated in the literature, this study highlights the antifungal activity of xanthohumol, extracted from leaves and cones, against another fungus, *V. inaequalis.* The obtention of low IC_50_ for xanthohumol, whether obtained from the leaves (1.6 and 5.1 mg·L^−1^) or from the cones (2.8 and 7.4 mg·L^−1^), against the two strains (S755 and rs552) used in this study, deserves to be underlined, especially since the IC_50_ of copper sulphate, used as a positive control, were higher against the two strains (194.6 mg·L^−1^ and 44.4 mg·L^−1^). Although xanthohumol appeared to be very active on *V. inaequalis*, the activity of fraction 4 suggests a potential interaction with other compounds. Many studies reported antifungal activities of xanthohumol, but the mode of action is still unknown. However, Yan et al. [27] studied the antifungal mechanism of isoxanthohumol, its corresponding flavanone, and suggested that it is related to metabolism by affecting the carbohydrate metabolic process, destroying the TCA cycle and blocking the generation of ATP by inhibiting respiration. Isoxanthohumol could also affect the membrane, inducing membrane lipid peroxidation.

## 5. Conclusions

Hop-growing generates agricultural waste because the leaves and stems remain under-exploited by-products. There is currently an increasing interest in the research for a potential valorisation of this biomass. It can indeed represent a source of bioactive compounds that could be interesting in the cosmetic, food, pharmaceutical industries, or even in plant protection. For decades, hop has been studied for its antimicrobial properties. In recent years, more and more studies have focused on the antifungal properties of hop against phytopathogenic fungi. The aim of this study was therefore to evaluate the potential antifungal activities of different parts of the hop plant (cones, leaves, stems and rhizomes) against the problematic fungus *Venturia inaequalis*. In vitro assays showed that leaf extracts were the most active extracts tested against two strains of *V. inaequalis* with different sensitivities to triazole fungicides. Thus, the dichloromethane sub-extract from the leaves was fractioned, and the fraction containing xanthohumol stood out. This chalcone purified from hop leaves appeared to be very active on spore germination of *V. inaequalis*. In addition, the strains seemed to have different sensitivities to hop metabolites; however, extracts of leaves and xanthohumol still have significant activities against both strains. In conclusion, hop extracts and xanthohumol show promise as botanical agents to control the phytopathogen *V. inaequalis*.

## Figures and Tables

**Table 1:** Comparison of strain sensitivity to two triazole fungicides and copper sulphate by analysis of their IC_50_ values.

Tested Substance	Strain	IC_50_ (mg·L^−1^)	Statistical Analysis	Difference between Strains
Tebuconazole	S755	0.009	a	Yes
	rs552	1.45	b	
Difenoconazole	S755	<0.0001	a	Yes
	rs552	0.06	b	
Copper sulphate	S755	194.6	b	Yes
	rs552	44.5	a	

**Table 2:** Comparison of strain sensitivities to hop extracts, fraction from leaf DSE and xanthohumol by analysis of their IC_50_. NA = Not Active; NC = Not Calculable.

Hop Parts	Extracts, Fractions or Purified Metabolite	Strains	IC_50_ (mg·L^−1^)	Statistical Analysis	Difference between Strains
Cones	CHE	S755	NC	-	Yes
		rs552	389.7	-	
	DSE	S755	NC	-	Yes
		rs552	361.6	-	
Leaves	CHE	S755	28.2	a	Yes
		rs552	62.5	b	
	DSE	S755	5.2	a	Yes
		rs552	10.5	b	
Stems	CHE	S755	59.3	a	Yes
		rs552	242.1	b	
	DSE	S755	15.6	a	Yes
		rs552	43.7	b	
Rhizomes	CHE	S755	NA	-	No
		rs552	NA	-	
	DSE	S755	NA	-	No
		rs552	NA	-	
Leaves	F1	S755	NA	-	No
		rs552	NA	-	
	F2	S755	NA	-	No
		rs552	NA	-	
	F3	S755	NA	-	No
		rs552	NA	-	
	F4	S755	0.25	a	Yes
		rs552	0.97	b	
	F5	S755	NC	-	Yes
		rs552	20.6	-	
	F6	S755	NA	-	No
		rs552	NA	-	
	F7	S755	NA	-	No
		rs552	NA	-	
Leaves	Xanthohumol 99%	S755	1.6	a	Yes
		rs552	5.1	b	

## Data Availability

The data presented in this study are available on request from the corresponding author.

## Supplementary Materials

The following supporting information can be downloaded at: https://www.mdpi.com/article/10.3390/microorganisms11061605/s1, Figure S1: Chromatograms by UPLC-UV-MS at 330 nm (method 1) of all fractions obtained from hop leaf DSE after preparative HPLC; Figure S2: Purity of xanthohumol in fraction F4 on the basis of PDA chromatogram (method 1); Figure S3: Chromatograms obtained by UPLC-UV-MS (method 1) of F4 (MaxPlot, 370 nm, TIC in positive mode, TIC in negative mode) and xanthohumol purified from hop cones (370 nm); Figure S4: Chromatograms obtained by UPLC-UV-MS (method 2) of xanthohumol purified from hop leaves (MaxPlot, 370 nm, TIC in positive mode, TIC in negative mode) as well as UV spectrum and mass spectrum; Figure S5: Purity of xanthohumol purified from hop leaves on the basis of PDA chromatogram (method 2); Figure S6: Chromatograms obtained by UPLC-UV-MS (method 3) of xanthohumol purified from hop cones (Max Plot, 370 nm, TIC in negative mode) as well as UV spectrum and mass spectrum.
