# Supplementary material for: Antifungal Activity of Hop Leaf Extracts and Xanthohumol on Two Strains of Venturia inaequalis with Different Sensitivities to Triazoles
Source: Microorganisms. 2023 Jun 17;11(6):1605. doi: 10.3390/microorganisms11061605 (PMC10300927; doi:10.3390/microorganisms11061605)
Supplement: Supplementary file 1 [file microorganisms-11-01605-s001.zip › microorganisms-2445732-supplementary.pdf]

Supplementary Materials

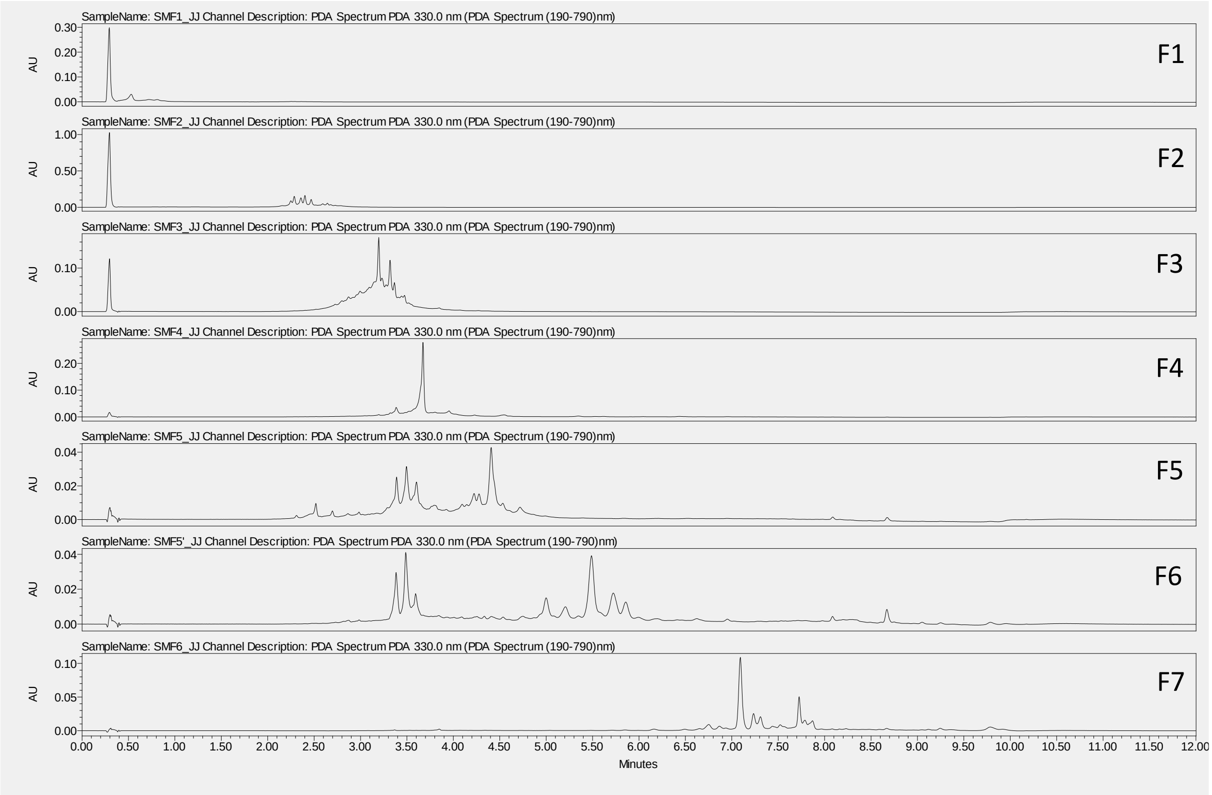

Figure S1. Chromatograms by UPLC-UV-MS at 330 nm (method 1) of all fractions obtained from hop leaf DSE after preparative HPLC

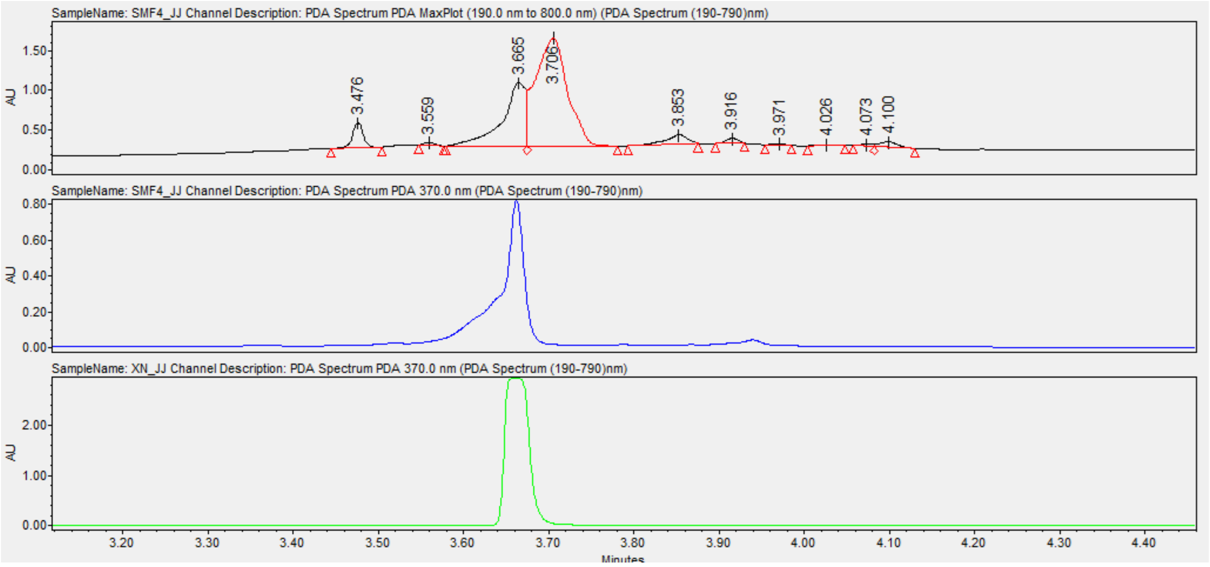

	Name	Retention Time (min)	Area (µV*sec)	% Area	Height (µV)	Signal_to_Noise_Value
1		3.476	267406	4.69	311523	
2		3.559	32132	0.56	36669	
3		3.665	1506435	26.42	791937	
4		3.706	3485673	61.12	1348596	
5		3.853	203307	3.57	116541	
6		3.916	61079	1.07	60890	
7		3.971	15216	0.27	15352	
8		4.026	14191	0.25	10791	
9		4.073	24516	0.43	21919	
10		4.100	92848	1.63	65388	

Figure S2. Purity of xanthohumol in fraction F4 on the basis of PDA chromatogram (method 1)

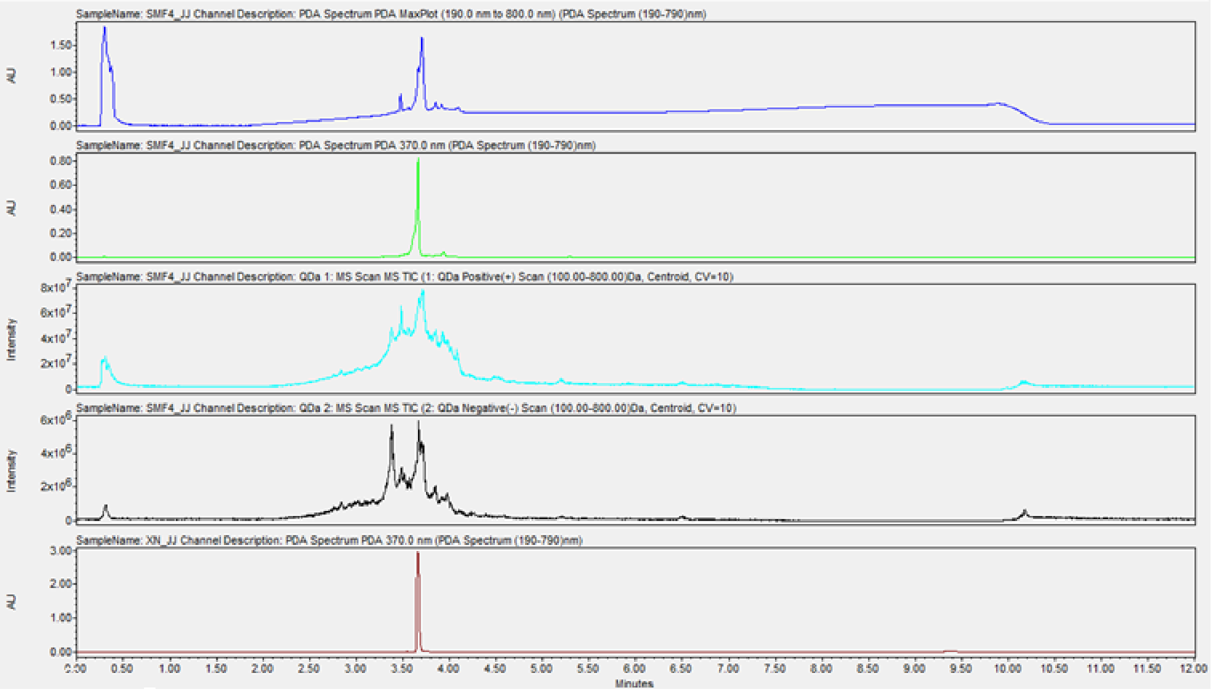

Figure S3. Chromatograms obtained by UPLC-UV-MS (method 1) of F4 (MaxPlot, 370 nm, TIC in positive mode, TIC in negative mode) and xanthohumol purified from hop cones (370 nm).

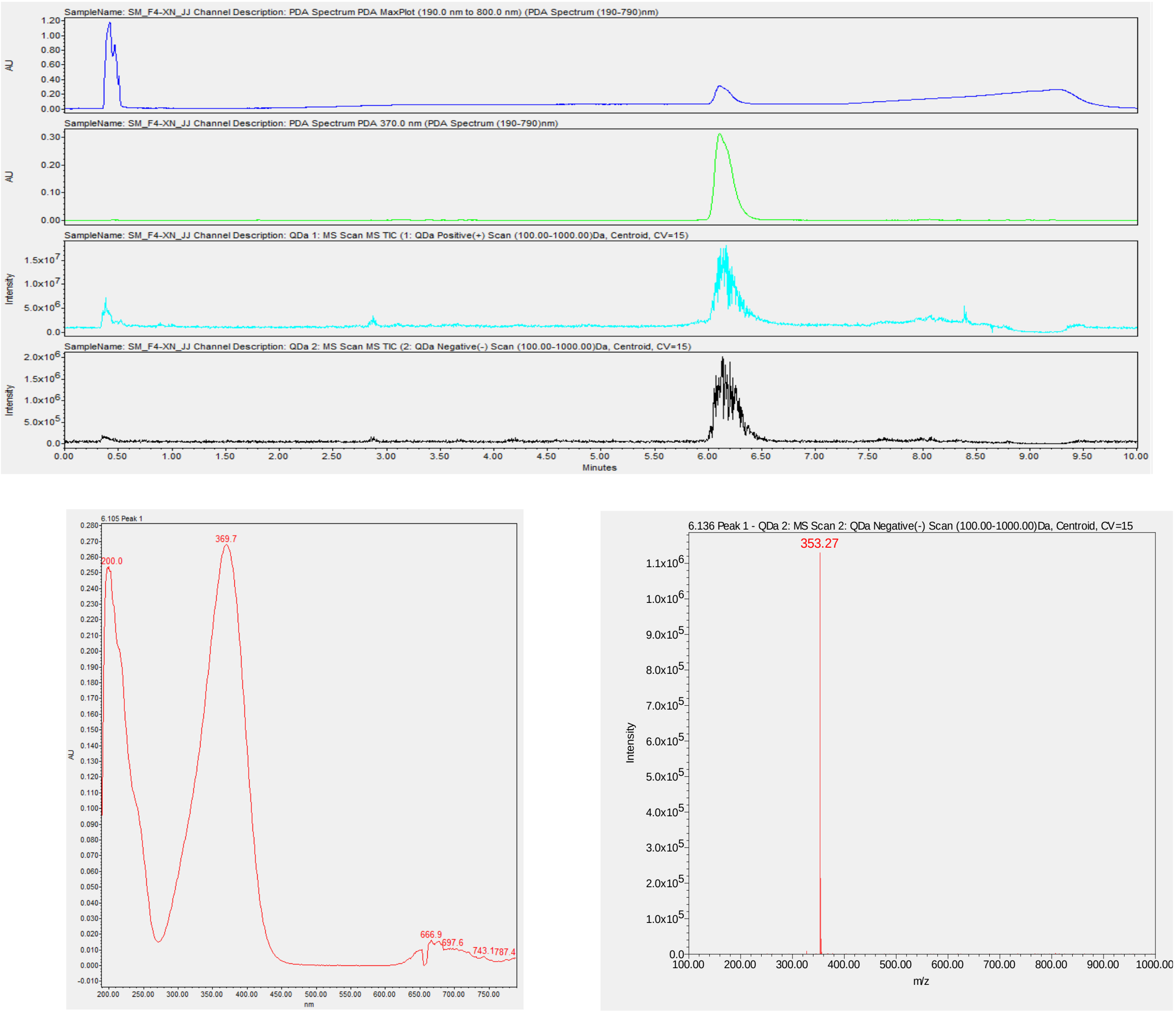

Figure S4. Chromatograms obtained by UPLC-UV-MS (method 2) of xanthohumol purified from hop leaves (MaxPlot, 370 nm, TIC in positive mode, TIC in negative mode) as well as UV spectrum and mass spectrum

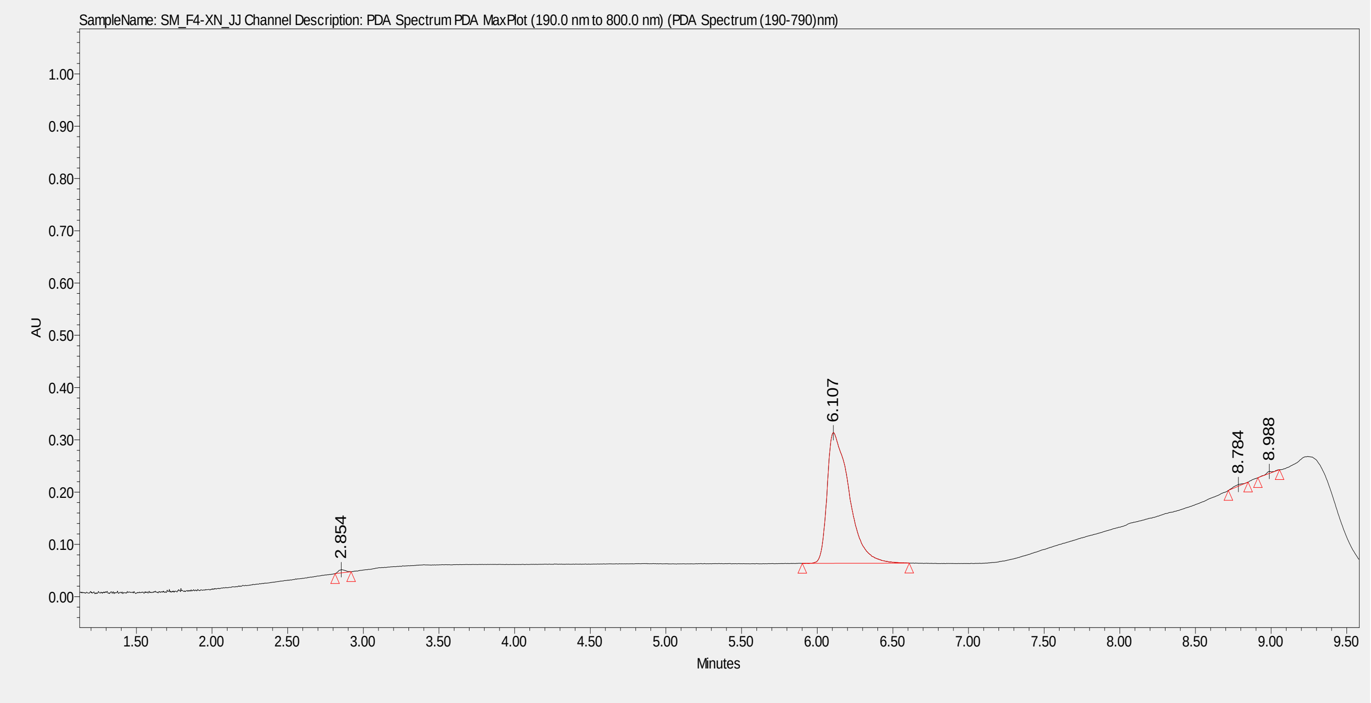

	Name	Retention Time (min)	Area (µV*sec)	% Area	Height (µV)	Signal_to_Noise_Value
1		2.855	9830	0.43	4246	
2		6.107	2261497	99.06	240441	
3		8.055	1079	0.05	843	
4		8.784	5745	0.25	2102	
5		8.989	4836	0.21	3055	

Figure S5. Purity of xanthohumol purified from hop leaves on the basis of PDA chromatogram (method 2)

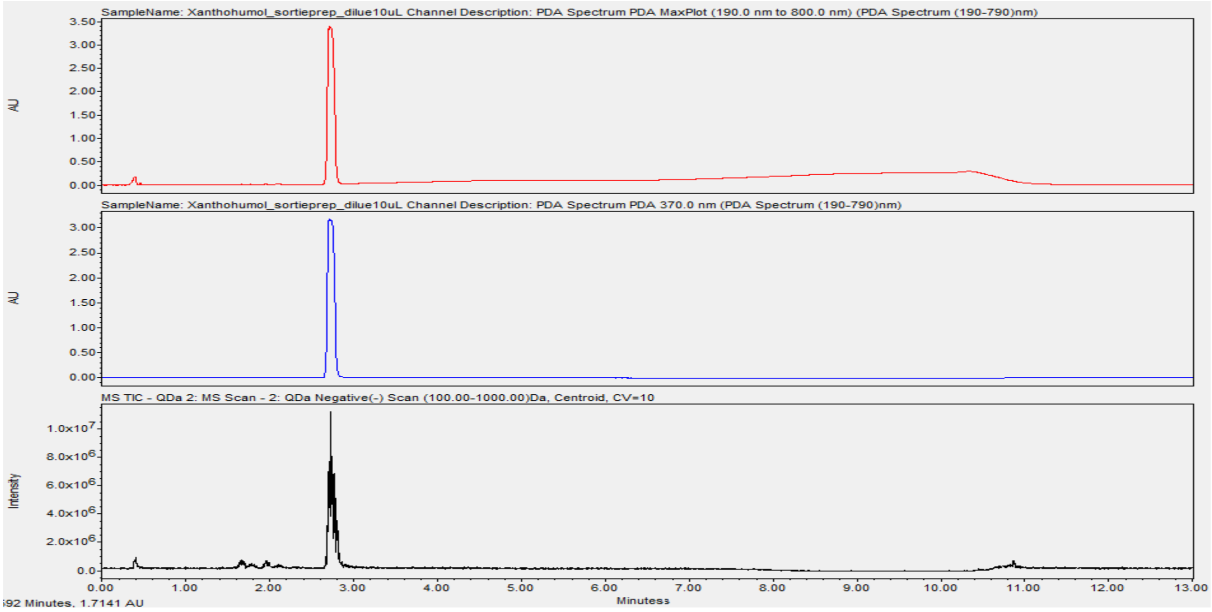

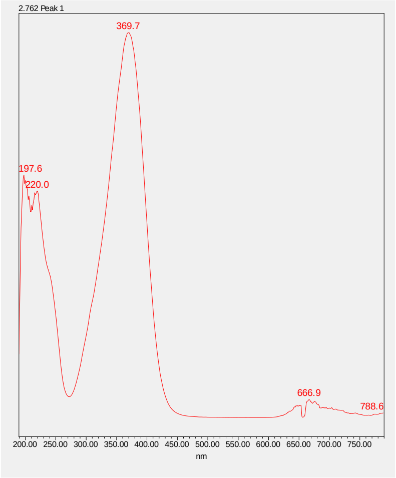

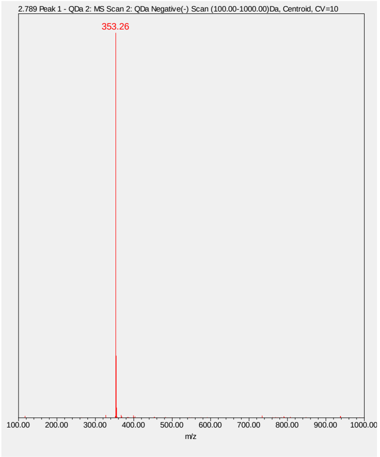

Figure S6. Chromatograms obtained by UPLC-UV-MS (method 3) of xanthohumol purified from hop cones (Max Plot, 370 nm, TIC in negative mode) as well as UV spectrum and mass spectrum
